# A cross-sectional study of keratoconjunctivitis among dairy cattle farms subject to Mediterranean climatic conditions

**DOI:** 10.1007/s11250-025-04341-7

**Published:** 2025-03-25

**Authors:** L. H. Maartens, P. N. Thompson, J. D. Grewar, J. Picard, B. Gummow

**Affiliations:** 1 Department of Production Animal Studies, Faculty of Veterinary Science, University of Pretoria, Pretoria, South Africa; 2 Discipline of Veterinary Science, James Cook University, Townsville, Australia

**Keywords:** Keratoconjunctivitis, Dairy cattle, *Moraxella*, Risk factors, Prevalence, Multivariable logistic regression

## Abstract

The epidemiology of bovine keratoconjunctivitis (BK) is complex and involves several conjunctival microbiota as well as host and environmental factors. Data about BK in dairy cattle are scarce, and even scarcer for dairy cattle in Mediterranean climates. We determined the BK prevalence among dairy cattle in Southern Africa’s Mediterranean climatic zone by examining 1,675 dairy cattle across 28 herds. Conjunctival swabs were analysed using real-time PCR. Risk factor data were collected by questionnaire and evaluated by multivariable logistic regression. The BK prevalence was 19.4%, (95% CI: 15.0 – 24.8%). *Mesomycoplasma bovoculi* was demonstrated in 75.7% of the sampled eyes (95% CI: 62.8 – 85.2%) across all farms. *Moraxella bovis* (18.3%, 95% CI: 11.9 – 27.0%) and *Moraxella bovoculi* (8.6%, 95% CI: 4.6 – 15.6%) were detected on 89% and 64% of the farms, respectively. *Mycoplasmopsis bovis* was rarely encountered. *Moraxella bovoculi* was significantly associated with BK (OR: 2.7, 95% CI: 1.3 – 5.6, Wald *P*: 0.01). Prevalence varied significantly by age and breed. Shade availability (OR: 2.5, 95% CI: 1.4 – 4.4, Wald *P* < 0.01), sanitation frequency, (OR: 0.4, 95% CI: 0.2 – 1.0, Wald *P*: 0.05), fly burden (OR: 2.1, 95% CI: 1.3 – 3.6, Wald *P* < 0.01) and introduced cattle (OR: 2.0, 95% CI: 1.1 – 3.5, Wald *P*: 0.02) were significantly associated with the prevalence of BK. Closed herds with proper biosecurity, fly control via sound management practices, and access to shade are recommended for preventing BK in dairy herds farmed in a Mediterranean climate.

**Supplementary Information:**

The online version contains supplementary material available at 10.1007/s11250-025-04341-7.

## Introduction

Keratoconjunctivitisis a common inflammatory condition of the bovine ocular surface (van Halderen and Henton 2004). It is characterized by mild to severe conjunctivitis that may progress to ulcerative keratitis (Angelos 2015). While the disease is typically self-limiting (O’Connor and Kneipp 2021), it is extremely painful, impacting on production and animal welfare (Woods et al. 2015; Williams 2010). Some cattle may suffer permanent blindness due to corneal perforation and disruption of intraocular homeostasis (Alexander 2010). Since the term “infectious bovine keratoconjunctivitis” (also known as “pinkeye” in lay terminology) is best used for instances of where keratoconjunctivitis occurs with high morbidity and rapid spread within a herd, this study will refer to the condition more generally as “bovine keratoconjunctivitis” (BK) (Maier et al. 2021; Kneipp 2021).

Until recently, BK was believed to be a contagious disease caused primarily by *Moraxella bovis* (*Mor. bovis*) (Loy et al. 2021b; Postma et al. 2008). This theory was supported by frequent isolations from affected eyes (Pugh 1969), the presence of virulence factors including type IV bacterial pili and an RTX-like cytotoxin (Beard et al. 1994; Pedersen et al. 1972), and the experimental reproduction of keratoconjunctivitis with certain *Mor. bovis* isolates (Chandler et al. 1979). The epidemiology of BK was believed to pivot around carrier animals and mechanical transmission by eye-frequenting insects, especially the European face fly (*Musca autumnalis*) (Brown and Atkins 1972; Seid 2019). While this may be partly true, many questions remain that cannot be explained by a model based on a single pathogen (O’Connor et al. 2021).

Later studies confirmed the presence of *Mor. bovis* in healthy and affected eyes, and the involvement of additional agents (Loy et al. 2021a; Schnee et al. 2015), such as *Moraxella bovoculi (Mor. bovoculi)* (Angelos 2010; O’Connor et al. 2012). *Moraxella bovis* and *Mor. bovoculi* may also coinfect the same eye. Unrelated bacteria, including *Mesomycoplasma bovoculi* (*Mes. bovoculi*) and *Mycoplasmopsis bovis* (*Myc. bovoculi*) (previously *Mycoplasma bovoculi* and *Mycoplasma bovis*, respectively) have also been reported from BK outbreaks (Gupta et al. 2018; Levisohn et al. 2004). Metagenomic analyses suggested that *Mor. bovis*, *Mor. bovoculi* and certain mycoplasmas, e.g. *Mes. bovoculi*, may be part of the normal bovine conjunctival microbiota (Bartenslager et al. 2021; Cullen et al. 2017). Hence the role of microbial factors in BK remains complex and is not yet fully understood.

Heifers (2—12 months) are at higher risk of developing BK than adult cows (Funk et al. 2014), while *Bos indicus* breeds have a lower risk than *Bos taurus* breeds (Frisch 1975; Gleeson 1965). Low peri-ocular pigmentation levels were initially implicated for the Hereford’s marked predisposition for BK (Snowder et al. 2005; Frisch 1975), but genetic studies identified additional genetic factors (Casas and Stone 2006; Kizilkaya et al. 2011; O’Connor 2021b). Host factors therefore influence the disease, and their role in dairy cattle requires further clarification.

The ocular surface is constantly exposed to injurious factors from the environment (Sack et al. 2001). The innate ocular defences and regenerative capacity of the corneal and conjunctival epithelium normally maintain homeostasis at the ocular surface, facilitating vision (Sack et al. 2001). Exposure to excessive quantities of injurious factors can overwhelm the eye’s defences and capacity for repair, leaving the ocular surface susceptible to colonisation by pathogenic microbes (Angelos et al. 2021). Seasonal trends in physical entities (e.g. ultraviolet radiation from the sun), mechanical irritants (e.g. dust) and biological factors (e.g. eye-frequenting insects) plausibly explained the seasonality of BK. (Kneipp et al. 2021; Maier et al. 2021; Seid 2019). Environmental factors are therefore an important component of the disease but their role in dairy farm management needs further investigation.

There remains a paucity of epidemiological information on BK in dairy cattle (O’Connor et al. 2021). Although many aspects of BK among beef cattle may be applicable, the metabolic demands of lactation and management factors associated with modern dairies may impose important differences (Vlasova and Saif 2021). In this study the prevalence of BK was investigated among dairy cattle in the Western Cape of South Africa, an area located in one of the world’s Mediterranean climatic zones (Polade et al. 2017), which experiences long dry summers that are conducive to BK. The study also explored farmer’s perceptions about BK and risk factors that could play a role in the epidemiology of BK in these dairy herds.

## Materials and methods

A cross-sectional study was conducted to estimate BK prevalence among dairy cattle, identify the associated risk factors and understand the dairy farmers’ perceptions about this condition. The study was conducted in the Western Cape Province of South Africa, in an area located between 33°S and 34.5°S latitude and 18°E and 21°E longitude within a Mediterranean climatic zone.

A multistage sampling approach was followed, with “herd” as the primary sampling unit and “dairy cattle” as the secondary. A preliminary sample size of ~ 130 dairy cattle was calculated using the formula (Martin et al. 1987):

$$n=\frac{{Z}^{2}\cdot \widehat{P}\cdot \widehat{Q}}{{L}^{2}}$$

where the critical value (*Z*) for a 95% confidence interval is 1.96; the prevalence of keratoconjunctivitis (*P̂*) estimated at 0.14; *Q̂* equalled 0.86 (calculated as 1—*P̂*); with an allowable error (*L*) of 0.06.

Limited resources for traveling and few respondents during the recruitment phase dictated a sampling strategy optimised for fewer clusters and larger cluster size. Given the time constraints between consecutive milkings and the average time required for effective animal restraint, clinical examination, and the collection of specimens, the maximum cluster size was estimated at 60 animals/farm.

Assuming a conservative intra-cluster correlation coefficient (ICC) for BK of 0.2 (Otte and Gumm 1997), and using the maximum cluster size, the preliminary sample size was adjusted by a design effect of 12.8 to account for the relative inefficiency of multistage sampling (Bennett et al. 1991). This implied a final sample size of at least 1664 cattle, rounded to 28 clusters with 60 cattle per cluster.

The paucity of participants precluded random selection of farms from a sampling frame. A convenience sample was collected from the available farms, which included the four dominant dairy cattle breeds in the region (Jersey, Holstein, Ayrshire and Guernsey) (Online Resource: Table S1), and a broad spectrum of age groups.

Both eyes of the 60 selected cattle in each herd were examined by a qualified veterinarian. The findings were recorded along with the animal’s identification number, breed, general health, and body condition. Digital images of each animal’s left and right eye were archived for quality control. The case definition for BK was based on the clinical signs of conjunctival and/or corneal inflammation. These changes ranged from minimal lacrimation without corneal involvement to advanced corneal ulceration, and eventual rupture of the eyeball, which were graded using a scoring system adapted from previous studies (Table 1) (George et al. 2005; Shugart et al. 1979). Eyes with minimal lacrimation and no corneal inflammation (eye score = 0.5) were excluded from the case definition, while eyes with copious lacrimation and tear marks extending to the mandible (eye score = 1.0) were included. Bovine keratoconjunctivitis cases were defined by the maximum eye score (max. eye score) between left and right.

**Table 1:** Scoring system for inflammatory changes in the conjunctiva and cornea

Description of eye lesion	Score
Healthy eye without visible lacrimation	0
Normal cornea, with minimal lacrimation confined to the medial canthus	0.5
Normal cornea, with marked lacrimation extending up to, or beyond, the mandible	1.0
Corneal oedema without a well-defined ulcer	1.5
Small corneal ulcer, less than 2 mm diameter	2.0
Medium corneal ulcer, 2 to 3 mm diameter	2.5
Large corneal ulcer, more than 3 mm diameter	3.0
Advanced corneal ulceration with descemetocele or keratoconus	3.5
Ruptured eye and blindness	4.0

Conjunctival swabs were taken in triplicate, from the conjunctival pouch between the lower and third eyelids and assessed in parallel. The first swab was kept on ice and submitted for PCR and the other two for bacterial isolation. DNA was extracted with the Roche Total Nucleic Acid Isolation Kit (Cat. no. 7658036001) on the automated Roche MagNA Pure 24 system. The primers and hydrolysis probes to detect *Mor. bovis*, *Mor. bovoculi*, *Myc. bovis* or *Mes. bovoculi* were synthesized by TIB Molbiol (www.tib-molbiol.de) using published sequences (Zheng et al. 2019). Modifying the hydrolysis probes for the available Roche instrumentation implied labelling of *Mor. bovis* and *Myc. bovis* probes with fluorescein amidite (FAM) and BHQ1, and the *Mor. bovoculi* and *Mes. bovoculi* probes with LightCycler® Red 610-N-hydroxysuccinimide ester (LC® Red 610) and BBQ. A minor modification was also made to the *Mes. bovoculi* probe by lengthening it with 6 base pairs for a slightly higher melting temperature (58 °C). The primer and probe sequences utilised for this study are available in the online resource: Table S2.

The assay was formulated as two duplex qPCR reactions, combining the reagents to detect *Mor. bovis* and *Mor. bovoculi* in the first subpanel and *Myc. bovis* and *Mes. bovoculi* in the second subpanel. All testing was done on the Roche LightCycler® Nano instrument, using amplification conditions comprising initial denaturing at 98 °C for 10 s; followed by 40 cycles denaturing at 95 °C for 10 s; and annealing and extension at 60 °C for 20 s. The reactions were conducted in 10 µl volumes, comprising 3.75 µl of the LightCycler® 480 RNA Master Hydrolysis Probes (Roche, REF 04991885001) reaction mixture, 1.6 µl PCR-grade water, 0.65 µl of activator, 1.0 µl of each primer and probe mixture and 3 µl sample DNA.

Positive controls were synthesised by GenScript (www.genscript.com), by cloning DNA fragments (80–130 bp) that encompassed the qPCR target regions on the chromosomal DNA of *Mor. bovis*, *Mor. bovoculi*, *Mes. bovoculi* and *Myc. bovis* into plasmid pUC57Simple. Based on the molecular weight of each plasmid, stocks were prepared containing 10^9^ copies of plasmid per µl. Tenfold serial dilutions were prepared from these plasmid stocks, for use in simplex and multiplex reactions to generate standard curves and determine the analytical sensitivity of each assay. The detection limit for each target was approximately 30 plasmid copies, although the probe concentration for *Mor. bovoculi* had to be increased threefold to achieve a similar sensitivity and acceptable amplification curves. Additional verifications were done by testing several *Mor. bovis* and *Mor. bovoculi* isolates from the Deltamune isolate collection.

For *Moraxella* isolation, 5% sheep blood agar was inoculated within 4 h after sample collection before transport to the laboratory. The inoculated plates were aerobically incubated at 37 °C for 24 to 72 h and examined daily for growth characteristics of *Moraxella* spp. (Juni and Bøvre 2015). Species identity was confirmed by Matrix-Assisted Laser Desorption/Ionization-Time of Flight mass spectrometry (MALDI-TOF) at AssureCloud (https://assurecloud.co.za). The mycoplasmas were cultivated at 37 °C in a selective growth medium, containing 1.12% Frey’s mycoplasma broth base, 1.85% brain heart infusion, 20% horse serum and 0.1% Azlocillin (Cook et al. 2016). The selective medium was inoculated on the farm and transported to the laboratory at ambient temperature. Subculturing was done after 3 weeks, into the same medium, and positive results were confirmed by demonstrating amplification with real-time PCR.

Farmer perceptions about BK, and the associated risk factors, were collected using a questionnaire (Online Resource: Questionnaire). The questionnaire covered data about disease trends during the five years preceding the study and environmental or management factors, such as flies and fly control, general sanitation, airborne dust levels, availability of shade, and the food sources utilised. Additional information was collected about vaccination against BK and respiratory pathogens.

The apparent prevalence (AP) of BK was calculated as the number of clinical cases per number of cattle sampled. The “svyset:” command in the Survey Data Analysis toolset of Stata®/BE 18.0 (Stata Statistical Software: Release 18. College Station, TX: StataCorp LLC.) was used to calculate the true prevalence with its 95% confidence interval, which adjusted for clustering and sampling weights.

The attribution of clustering to the overall sample variance was evaluated by calculating the intra-cluster correlation coefficient (ICC) according to the following formula (Fleiss et al. 2003):

$$\rho =\frac{\sum_{i=1}^{K}\left\{{Y}_{i+}\left({Y}_{i+}-1\right)-2P\left({n}_{i}-1\right){Y}_{i+}+{n}_{i}\left({n}_{i}-1\right){P}^{2}\right\}}{{\sum }_{i=1}^{K}\{{n}_{i}({n}_{i}-1)P(1-P)\}}$$

where *ρ* represents the ICC, *K* the number of participating farms*,* Y_i+_ the number of animals with BK in herd *i*, *n*_*i*_ the number of animals clinically examined in herd *i*, and *P* the apparent prevalence.

The clinical findings, laboratory results and questionnaire information were linked by farm and individual animal in a database managed on Epi Info™, version 7.5.2.0 (CDC, Atlanta GA, USA). Risk factors were univariately screened for potentially significant associations, by calculating odds ratios and Chi-square statistics in Microsoft Excel (Microsoft® Excel® for Microsoft 365 MSO, Version 2402). Risk factors were selected for further analysis in a mixed-effects multivariable logistic regression model when the Chi-square test yielded *P*-values < 0.2. The Fisher’s exact test was used instead of Chi-square, where risk factor data contained values < 5 in one or more of the contingency table quadrants. The mixed-effects logistic regression model was constructed using Stata®BE 18. Single independent variables were added in a stepwise hierarchical fashion while removing variables with Wald *P* > 0.05. Farm was included as a random effect, and adjustment for sampling weights was done using the “svy:” command. Potential interactions between *Mes. bovoculi* and the *Moraxella* spp. or *Myc. bovis* were evaluated by including composite variables in the analyses.

## Results

The participating farms varied in breed composition, management style, feeding strategy and herd size (Table 2). The combined population of cattle on these farms comprised ~ 45,400 animals, comprising 52% Holstein, 39% Jersey, 7% Ayrshire, 1% Guernsey and 1% mixed-breed cattle, which aligned well with current estimates of the dairy cattle breed composition in the region. (Online resource: Table S1). Twenty farms were managed semi-intensively, utilising pastures as well as total mixed rations or concentrates plus long-stemmed roughage. Pasture utilisation was seasonal on some of these farms, while others utilised it for specific production groups (e.g., post-pubertal heifers). The other eight farms were managed intensively, maintaining the lactating cows under roofed structures, and feeding a total mixed ration. Dry cows and heifers were typically kept in open pens comparable to feedlots on these farms. Herd sizes ranged from 141 to 11,152 (median: 874), being considerably larger on the intensively managed farms.

**Table 2:** Summary of the farm profiles and vaccination status

Dominant breed	Management style	Herd size:		Vaccinated herds
		*n* ≤ 874	*n* > 874	
Jersey	Semi-intensive	10	4	4 / 14 herds
	Intensive	-	1	0 / 1 herd
Holstein	Semi-intensive	-	1	0 / 1 herd
	Intensive	-	6	5 / 6 herds
Ayrshire	Semi-intensive	3	-	0 / 3 herds
	Intensive	-	1	1 / 1 herd
Guernsey	Semi-intensive	1	-	0 / 1 herd
	Intensive	-	-	-
Mixed breed	Semi-intensive	1	-	1 / 1 herd
	Intensive	-	-	-

Jersey cattle, which collectively made up 39% of the total cattle population on the participating farms, predominated on the semi-intensively managed farms. Holsteins (comprising 52% of the cattle on the participating farms) were more commonly used on the intensively managed farms. Of the four Ayrshire herds, one was managed intensively and the others semi-intensively. The single Guernsey herd and a recently established herd comprising mixed-breed animals were both managed semi-intensively.

Sanitation practices varied between the participating farms. Most farms had concrete flooring around the milking parlour that were hosed-off after every milking or once daily. On 15% of the farms, however, manure was left around the parlour for periods exceeding one week. The cleaning of holding pens varied according to management style, with intensively managed farms being cleaned more often. Most farmers (43%) removed manure every 2 to 6 months from the pens, while some (15%) removed the manure with intervals ≤ 7 days (Online Resource: Figure S1).

Fly control programs varied, usually incorporating one or more of the following measures: (i) insecticides and repellents on animals or nearby structures, (ii) fly traps of varying designs, (iii) insect growth regulators and (iv) predatory or parasitic wasps. Some farmers specified the benefit of (v), reducing breeding substrates via effluent treatment plants, or (vi) the use of backyard chickens feeding on fly larvae and pupae.

Approximately 39% of the participants immunised their herds against BK using Piliguard® Pinkeye-1 Trivalent, MSD Animal Health, (Reg no G2803, Act 36/1947). The vaccine was mostly administered to pre-pubertal heifers around weaning age, although some farmers immunised the whole herd (Online resource: Figure S2). Many of the participants vaccinated their herds against bovine respiratory diseases (*e.g.*, bovine herpes virus I, bovine respiratory syncytial virus, etc.). No associations were detected between BK and vaccination against pinkeye or against the bovine respiratory disease complex.

Of the 1675 examined cattle, 270 animals had a max. eye score ≥ 1.0 (Table 3), which implied an apparent BK prevalence of 16.1% (95% CI: 14.4—18%), and a true prevalence of 19.4% (95% CI: 15 – 24.8%) after accounting for clustering and sampling weights. The ICC was calculated to be 0.037.

**Table 3:** Summary of the maximum eye scores recorded for 1675 dairy cattle

Max score:	Interpretation	*n*	Apparent prevalence %	(95% CI)
0	Healthy	769	45.9	(43.5 – 48.3)
0.5	Healthy	636	38.0	(35.7 – 40.3)
1	Diseased	126	7.5	(6.4 – 8.9)
1.5	Diseased	37	2.2	(1.6 – 3.0)
2	Diseased	49	2.9	(2.2 – 3.9)
2.5	Diseased	16	1.0	(0.6 – 1.6)
3	Diseased	26	1.6	(1.1 – 2.3)
3.5	Diseased	12	0.7	(0.4 – 1.3)
4	Diseased	4	0.2	(0.1 – 0.6)

All four bacterial agents were demonstrated on conjunctival swabs via real-time PCR or bacterial isolation. *Mesomycoplasma bovoculi* was highly prevalent and occurred on all the farms, with 75.7% (95% CI: 62.8 – 85.2%) of the sampled eyes testing positive. *Moraxella bovis* and *Mor. bovoculi* were also demonstrated on most of the farms. *Moraxella bovis* occurred more frequently, with 25/28 farms having the organism and 18.3% (95% CI: 11.9 – 27.0%) of the sampled eyes testing positive. *Moraxella bovoculi* was detected on 18/28 farms, while 8.6% (95% CI: 4.6 – 15.6%) of the sampled eyes tested positive. *Mycoplasmopsis bovis* was only detected on 5/28 farms, and in only 0.2% (95% CI: 0 – 0.7%) of the sampled eyes (Online resource: Table S3).

Bovine keratoconjunctivitis was detected among all age and production groups (Table 4). The group prevalence ranged from 6.6% (95% CI: 0 – 13.2%) among dry cows, to 33.5% (95% CI: 23.7 – 43.3) among pre-weaned calves. Of the 30 pre-weaned calves classified as BK-positive, only two animals had corneal involvement. The apparent prevalence of BK varied according to breed. From the three dominant breeds in the area, Holsteins (21.7%, 95% CI: 13.7 – 29.8) showed the highest prevalence, followed by Ayrshires (18.4%, 95% CI: 12.9 – 23.8) and Jerseys (12.9%, 95% CI: 9.5 – 16.3). A combined category for minor breeds, including Guernseys and mixed-breed cattle, showed a similar prevalence to that of Ayrshires.

**Table 4:** Maximum eye scores and the BK prevalence (%) in dairy cattle of different age and production groups

Group	*n*	Maximum Eye Scores (%)										
		BK absent		BK present							Prevalence (%)^*^ (95% CI)	
		0	0.5	1	1.5	2	2.5	3	3.5	4		
Pre-weaned calves	109	37.6	34.9	25.7	0.0	0.0	0.0	0.0	1.8	0.0	33.5	(23.7 – 43.3)
Pre-pubertal heifers	356	45.8	35.1	9.6	2.8	2.8	1.4	1.1	1.4	0.0	19.9	(13.8 – 26.1)
Post-pubertal heifers	148	44.6	35.8	6.1	4.7	4.7	0.0	2.7	0.7	0.7	14.1	(5.3 – 23.0)
First lactation heifers	223	41.7	40.8	4.0	2.7	5.4	2.7	1.8	0.0	0.9	27.6	(20.2 −35.1)
Lactating cows	727	47.3	40.0	5.4	1.7	2.5	0.7	1.9	0.6	0.0	14.5	(11.2 – 17.9)
Dry cows	112	55.4	33.9	6.3	1.8	1.8	0.0	0.0	0.0	0.9	6.6	(0 – 13.2)

^*^Adjusted for clustering and sampling weights

*BK* bovine keratoconjunctivitis

Forty-nine independent variables comprising several agent, host and environmental factors were screened during univariate analysis for potential relationships with BK (Online Resource: Table S4). Thirty-six of these variables (Table 5) were selected, based on *P* < 0.2, for inclusion in the mixed-effects logistic regression model. Several factors were significantly associated with BK in the final model (Table 6). Pre-weaned calves, followed by first lactation heifers, had the highest odds of BK and dry cows the lowest. Of the local breeds, Jerseys had the lowest odds, while mixed-breed cattle and Guernseys had the highest. Higher odds of BK were found among cattle from herds (i) being sampled when the perceived fly burden was high, (ii) containing recently introduced cattle, and (iii) being maintained without sufficient shade. An inverse association was found between BK and (iv) the use of backyard chickens for fly control, and farms where (v) holding pens were cleaned with intervals ≤ 7 days. Of the agent factors evaluated, only *Mor. bovoculi* had a significant association with BK.

**Table 5:** Univariate analysis of the associations between BK and potential host, environmental and agent risk factors

Variables and category	Cattle exposed		Cattle with BK	Odds ratio	95% CI	*P*-value
Breed:						< 0.01^a^
Holstein	405		88	1.9	1.4 – 2.5	
Jersey	944		122	(base)		
Guernsey	39		5	1.0	0.4 – 2.6	
Ayrshire	256		47	1.5	1.0 – 2.2	
Mixed breed	31		8	2.3	1.0 – 5.4	
Production group:						< 0.01^a^
Lactating cows	727		92	1.2	0.6 – 2.3	
Dry cows	112		12	(base)		
First lactation heifers	223		39	1.8	0.9 – 3.5	
Post-pubertal heifers	148		29	2.0	0.9 – 4.2	
Pre-pubertal heifers	356		68	2.0	1.0 – 3.8	
Pre-weaned calves	109		30	3.2	1.5 – 6.6	
Sampling month:						0.08^a^
January	238		36	0.8	0.5 – 1.2	
February	359		58	0.9	0.6 – 1.2	
March	778		141	(base)		
April	300		35	0.6	0.4 – 0.9	
State vet. District:						0.13^a^
Boland	179		21	0.7	0.4 – 1.1	
Malmesbury	299		59	1.3	0.9 – 1.8	
Swellendam	958		154	(base)		
Worcester	239		36	0.9	0.6 – 1.4	
Animals in lactation	950		131	0.7	0.5 – 0.9	< 0.01^a^
High dust levels 1 month before sampling	898		164	1.4	1.1 – 1.8	0.01^a^
High dust levels during month of sampling	598		118	1.5	1.1 – 1.9	< 0.01^a^
High fly burden 1 month before sampling	959		169	1.3	1.0 – 1.7	0.05^a^
High fly burden during month of sampling	599		128	1.8	1.4 – 2.3	< 0.01^a^
Fly control by backyard chickens	120		6	0.3	0.1 – 0.6	< 0.01^a^
Fly control by insect growth regulators	360		75	1.5	1.1 – 2.0	0.01^a^
Fly control by pesticides	1377		237	1.7	1.1 – 2.5	0.01^a^
Fly control by parasitic wasps	119		9	0.4	0.2 – 0.8	0.01^a^
No fly control program	59		5	0.5	0.2 – 1.2	0.15^b^
Ration containing apple pomace	231		29	0.7	0.5 – 1.1	0.11^a^
Ration containing citrus residue	461		60	0.7	0.5 – 1.0	0.03^a^
Ration containing silage	733		98	0.7	0.5 – 0.9	0.01^a^
Ration containing dry hay	1087		178	0.6	0.5 – 0.8	< 0.01^a^
Ration containing grains (meal or pellets)	1034		145	0.7	0.5 – 0.9	< 0.01^a^
Grazing on irrigated pastures	832		123	0.8	0.6 – 1.1	0.14^a^
Utilizing crop residues	249		31	0.7	0.5 – 1.1	0.09^a^
Larger herds (≥ 874)	838		151	1.3	1.0 – 1.7	0.03^a^
Higher stocking density (≥ 40 animals/ha)	359		71	1.4	1.0 – 1.9	0.03^a^
Stable herd size during last 5 years	1017	145		0.7	0.5 – 0.9	0.01^a^
Cattle introductions during the last 5 years	419	89		1.6	1.2 – 2.1	< 0.01^a^
Cleaning frequency, milking area: ≤ 7 days	1436	251		2.5	1.5 – 4.0	< 0.01^a^
Cleaning frequency, holding pens: ≤ 7 days	239	20		0.4	0.3 – 0.7	< 0.01^a^
Gravel flooring around milking parlour	418	56		0.8	0.5 – 1.0	0.08^a^
Insufficient shade in camps	756	147		1.6	1.2 – 2.0	< 0.01^a^
Access to dappled shade under trees	533	72		0.7	0.6 – 1.0	0.05^a^
Vaccinating against pinkeye (heifers only)	299	61		1.4	1.0 – 2.0	0.03^a^
Using inactivated BHV1, BVD vaccines	538	100		1.3	1.0 – 1.7	0.06^a^
Using live BVD vaccines	538	69		0.7	0.5 – 0.9	0.01^a^
*Mor. bovis* present	259	56		1.5	1.1 – 2.2	0.01^a^
*Mor. bovoculi* present	138	40		2.3	1.6 – 3.4	< 0.01^a^
*Mes. bovoculi* present	1353	239		2.0	1.4 – 3.0	< 0.01^a^

Only factors with *P* ≤ 0.2 qualifying for multivariable analysis are shown

*BK* Bovine keratoconjunctivitis, *BRD* Bovine respiratory disease, *BHV1 *Bovine herpesvirus type 1, *BVD* Bovine viral diarrhea

^a^*P*-value derived by Chi-squared analysis

^b^*P*-value derived by Fisher’s exact test

**Table 6:** Analysis of the associations between BK and risk factors in a mixed-effects multivariable logistic regression model

Variable and category	Odds ratio	95% CI	Wald *P* -value
Production group			
Lactating cows	2.2	0.6 – 7.7	0.20
Dry cows	(base)		
First lactation heifers	5.2	1.4 – 18.7	0.01
Post-pubertal heifers	1.9	0.7 – 5.4	0.23
Pre-pubertal heifers	3.7	1.1 – 12.8	0.04
Pre-weaned calves	7.0	1.5 – 33.7	0.02
Breed			
Holstein	3.7	0.4 – 33.5	0.24
Jersey	(base)		
Guernsey	10.9	4.1 – 29.5	< 0.01
Ayrshire	2.1	0.6 – 7.0	0.21
Mixed	4.7	1.5 – 15.0	< 0.01
High fly burden during month of sampling	2.2	1.3 – 3.6	0.01
Cattle introductions during the last 5 years	2.0	1.1 – 3.5	0.02
Fly control by backyard chickens	0.1	0.0 – 0.4	< 0.01
Insufficient shade in camps	2.5	1.4 – 4.4	< 0.01
Cleaning frequency of the pens ≤ 7 days	0.4	0.2 – 1.0	0.05
Mor. bovoculi present	2.7	1.3 – 5.6	0.01

*BK* Bovine keratoconjunctivitis

**Fig. 1:**
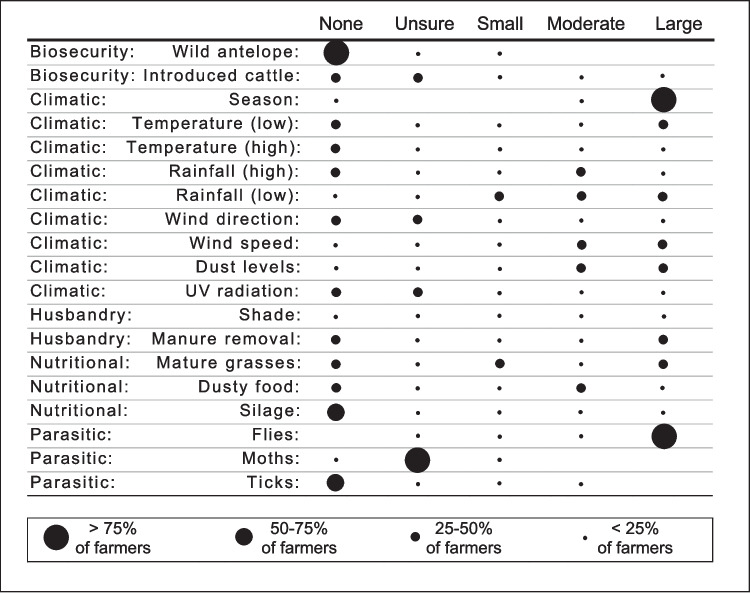
Summary of the farmer’s perceptions about the contribution of risk factors to bovine keratoconjunctivitis in their herds

Most participants perceived age, but not breed, as an important host factor for BK. Pre-pubertal heifers were perceived to have the highest risk, while pre-weaned calves were seldom affected according to their perception. Biosecurity and contact with other bovine or non-bovine animals (*e.g.* wild antelope) were not perceived as important contributors to BK (Fig. 1).

**Fig. 2:**
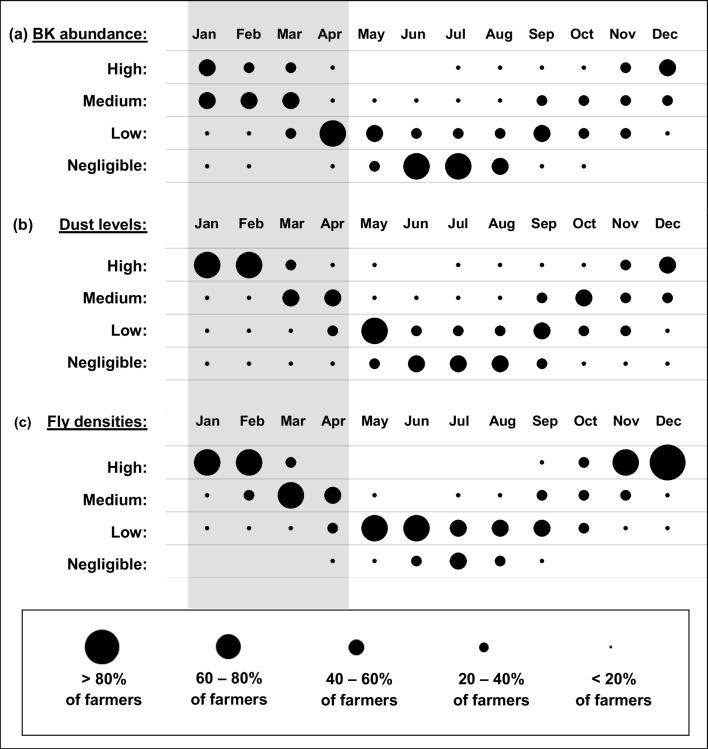
Farmers’ perceptions about the seasonal trends of (**a**) the relative abundance of bovine keratoconjunctivitis (BK) in their herds, (**b**) ambient dust levels, and (**c**) intensity of the fly burden on their farms. The size of the dots reflects the number of concurring answers about BK levels for a given month. The grey window represents the months during which this study was performed

With few exceptions, the participants considered season to be a major risk factor for BK (Fig. 1), with peak levels of disease expected during the first half of summer (Fig. 2a). Perceptions differed regarding the importance of the various meteorological factors (Fig. 1). Low rainfall, high wind speed and dust were generally viewed as important, with the highest dust levels expected towards the middle of summer (Fig. 2b). While farmers had opposing views about the role of low temperatures, high temperatures, UV radiation and wind direction were not perceived as major contributors to BK. (Fig. 1).

The participants had divergent perceptions about the role of shade, manure removal and grass awns (Fig. 1). While some perceived grass awns to be a risk, many remarked that their nutritional programs precluded exposure to seeding grasses. Dusty food was considered moderately important by some, while silage was not perceived to be a risk factor for BK.

Eye-frequenting flies were perceived to be a major contributor to BK (Fig. 1), and many ascribed the seasonality of BK to high fly densities occurring in summer (Fig. 2c). Other parasitic factors, such as ticks, were not perceived to be important, while lachryphagous moths were largely unknown to the participants.

## Discussion

Dairy production presents a major agricultural activity in Mediterranean climatic zones, including the Mediterranean basin, large areas of California and regions in southern Australia, southwestern South America and the southwestern corner of Africa. The low rainfall, high dust levels, and intense solar radiation of the harsh Mediterranean summers present a challenging environment for ocular homeostasis (Polade et al. 2017). As climatic change progresses the harsh Mediterranean summer conditions are intensifying with soaring temperatures, longer dry seasons and accelerated erosion (Urdiales-Flores et al. 2023).

Dairy production is labour intensive and recruiting volunteers among commercial producers, for research purposes, will always be challenging. Careful planning of the recruitment phase and incorporating innovative ways to stimulate interest will be paramount for future studies in this sector. In the case of this study, several factors (including biosecurity concerns associated with foot and mouth disease) placed additional constraints on the recruitment process, necessitating modification of the sampling strategy. Using a two-stage approach with large cluster sizes and comparatively few clusters, the summer prevalence of BK among Western Cape dairy cattle was estimated to be 19.4%. The precision of the estimate was slightly greater than originally intended, due to the fact that the degree of clustering was lower than expected (ICC = 0.037). While this information can also be relevant to dairy cattle in other Mediterranean climatic zones, it should be emphasized that BK prevalence is likely to be lower during the wet season.

While *Mor. bovis* and *Mor. bovoculi* were both found in healthy and affected eyes of dairy cattle in this study, only *Mor. bovoculi* showed a significant association with BK. Although *Moraxella bovoculi* is often more prevalent in affected eyes, its role in causing BK remains unclear (O’Connor et al. 2021; Schnee et al. 2015). Since BK could be experimentally reproduced with *Moraxella bovis* only (Loy et al. 2021b; O’Connor 2021a), this agent is still considered the dominant microbial factor. However, *Moraxella. bovoculi* shares some virulence factors with *Mor. bovis*, including type IV bacterial pili and an antigenically distinct RTX-like cytotoxin (genotype 1 strains). Considering the proven ability of *Mor. bovoculi* to recombine with other *Moraxella* species, its role in BK should not be underestimated (Dickey et al. 2018).

*Mesomycoplasma bovoculi* occurs commonly in the bovine conjunctiva and like *Moraxella s*pecies, this agent can be demonstrated in healthy and affected eyes (Schnee et al. 2015). While its presence in healthy eyes suggests a commensalistic relationship with cattle, *Mes. bovoculi* may enhance the effects of pathogens such as *Myc. bovis* and *Mor. bovis* (Friis and Pedersen 1979; Levisohn et al. 2004). In this study, *Mes. bovoculi* was abundantly present in healthy and diseased eyes. Considering its potential to aggravate the effects of pathogens, composite variables representing coinfections between *Mes. bovoculi* and each *Moraxella* species were evaluated in the multivariable logistic regression model, but these variables were not significantly associated with BK.

*Mycoplasmopsis bovis* is commonly associated with bovine mastitis, pneumonia, and arthritis (Maunsell et al. 2011). This agent is, however, pantropic in cattle and was previously recovered from affected eyes during BK outbreaks (Alberti et al. 2006; Levisohn et al. 2004). In this study, *Myc. bovis* was rarely detected and did not significantly contribute to BK.

Given the small number of herds (*n* = 28), the study might have lacked the statistical power to reliably detect all potential associations between BK and the risk factors influencing disease prevalence across different herds. This implies a higher likelihood of obtaining false negative results for risk factor associations, rather than false positives. Therefore, we can only report the significant associations that were detected, while the failure to detect an association does not necessarily imply the absence of a meaningful relationship. The risk factors identified in this study, including age, breed, availability of shade, high fly densities, certain fly control measures, adequate sanitation, and appropriate biosecurity, are consistent with published information about the secondary determinants of BK (Kneipp et al. 2021; Maier et al. 2021).

Age is a significant risk factor for BK (Dennis and Kneipp 2021). We confirmed that heifers (≤ 26 months) were significantly more affected than adult cows. The prevalence of BK was highest among pre-weaned calves (≥ 3 months). However, BK presented mainly as conjunctivitis and lacrimation in these calves. The scarcity of corneal ulcers in these young calves (Table 4), probably explains the farmers’ perception that pre-weaned calves rarely develop BK.

We found that Jerseys were less affected by BK than the other dairy breeds. Considering the well pigmented facial skin and eyelids of the Jersey, periocular pigmentation levels should be included in future assessments of risk factors for BK in dairy cattle.

Shade is an important welfare consideration for dairy cattle, especially in hot tropical and subtropical climates (Silanikove 2000). While adequate shade is crucial to optimise dairy production (Muller et al. 1994; Fournel et al. 2017; Kamal et al. 2018), we demonstrated that the increased availability of shade is associated with a reduction in the prevalence of BK. Most farms provided ample shade for pre-weaned calves, but limited access to shade likely increased the risk of BK among older heifers and cows on many farms. Providing access to shade for all reproductive groups can improve dairy cattle welfare and reduce the prevalence of BK.

We demonstrated significant associations between BK and three variables that directly, or indirectly, relate to fly abundance: (i) *High fly burden during month of sampling*, (ii) *cleaning frequency of the pens* ≤ *7 days*, and (iii) *fly control by backyard chickens*. These observations support the role of lachryphagous flies as risk factors for BK. Like the European face fly, regional face fly species (*e.g., M. lusoria* and *M. nevilli*) are equipped with elongated prestomal teeth that may abrade the conjunctiva (Brown and Adkins 1972; Kneipp et al. 2021; Snowder et al. 2005). The shorter prestomal teeth of other eye-frequenting *Muscidae, e.g.,* the common housefly (*M. domestica*), and the African face fly (*M. xanthomelas*), are less likely to abrade the ocular tissues (Broce and Elzinga 1984; Neville 2004). However, all *Musca* species regurgitate crop contents during feeding and grooming, facilitating pathogen transmission (Glass and Gerhard 1983, 1984; Stoffolano 2019).

Since flies breed in bovine dung, manure accumulation in holding pens enhances the fly burden. Sanitation frequency varied in our study area. The larger intensively managed farms cleaned their pens daily, via automated systems. Without automation, daily sanitation is not feasible. On semi-intensively managed farms, pens were cleaned weekly to monthly, while longer cleaning intervals (e.g., every 2 – 6 months) were common practice. Some farms never removed manure from the pens, or only during excessively wet conditions. Given that the muscid life cycle may be as short as ~ 1 week; sanitation intervals ≤ 7 days may theoretically decrease the fly burden (Moon 2018). This explains the lower prevalence of BK on farms with pen sanitation intervals ≤ 7 days, in our study.

Several farmers reported the use of backyard chickens under “other” fly control measures. When analysed as a risk factor, an unexpected reduction in BK prevalence was observed with the use of backyard chickens. This finding needs further investigation, since control by backyard chickens was not specifically included in the questionnaire.

According to traditional views, BK developed when flies transmitted *Mor. bovis* from symptomatic and asymptomatic carriers to naïve cattle (Hall 1984; Brown et al. 1998). Recent research, however, suggests that *Mor. bovis* and other potentially pathogenic microbes exist as commensals in the conjunctival microbiota, becoming pathogenic under certain conditions (Cullen et al. 2016; Bartenslager et al. 2021; Overstreet and Lotz 2016). In this study, we found a higher prevalence of BK among open herds with a history of recent cattle introductions. While this finding could easily be explained by traditional views, the introduction of novel microbial agents could also disturb the conjunctival microbiota. The association between open herds and BK should therefore not be regarded as contradictory to current epidemiological views.

Although dust is commonly cited as a risk factor for BK, we did not find a significant association between BK and the perceived dust levels on the farms. This may be due to the unusual amount of summer rain that fell in the area, which significantly reduced the animal’s exposure to dust.

The farmer perceptions recorded in this study should be interpreted with caution. The low response rate during recruitment also limited the number of participating farmers, impacting on statistical power and affecting the reliability of the findings. Additionally, asking animal owners to reflect on past observations may introduce recall bias, further influencing the results. Despite these limitations, the data on farmer perceptions may still be helpful to identify misconceptions about the disease and deficiencies in husbandry practices, which could be addressed in holistic control programs.

## Conclusion

While the primary determinant of this condition remains unknown, this study provided valuable epidemiological information regarding BK in dairy cattle. The clinical observations of this study showed that BK can be highly prevalent among dairy cattle in regions with a Mediterranean climate, such as the Western Cape of South Africa. The study also found a significant association between BK and *Mor. bovoculi,* one of several BK-associated agents forming part of the bovine conjunctival microbiota. Maintaining closed herds with proper biosecurity, controlling the fly burden via sound management practices, and ensuring access to shade is recommended in the prevention of BK on dairy farms.

## Supplementary Information

Below is the link to the electronic supplementary material.

Supplementary file1 (DOCX 1525 KB)

## Data Availability

The datasets applicable to this study will be archived via the James Cook University research data repository “Research Data JCU”, (https://research.jcu.edu.au/data/default/rdmp/home).
