# Supplementary material for: A cross-sectional study of keratoconjunctivitis among dairy cattle farms subject to Mediterranean climatic conditions
Source: Trop Anim Health Prod. 2025 Mar 25;57(3):141. doi: 10.1007/s11250-025-04341-7 (PMC11937216; doi:10.1007/s11250-025-04341-7)
Supplement: Supplementary file 1 — Supplementary file1 (DOCX 1525 KB) [file 11250_2025_4341_MOESM1_ESM.docx]

**A cross-sectional study of keratoconjunctivitis among dairy cattle farms subject to Mediterranean climatic conditions**

L. H. Maartens, P. N. Thompson, J. D. Grewar, J. Picard and B. Gummow

# **ONLINE RESOURCE**

**Questionnaire**

| \| **PINKEYE INVESTIGATION IN DAIRY CATTLE IN THE WESTERN CAPE** \| \| --- \| \| Conducted by: Dr Louis Maartens \| \| (A collaborative project between Deltamune Pty Ltd, University of Pretoria and James Cook University) \| |
| --- | --- | --- | --- |

|  | | |
| --- | --- | --- |
| **What is "Pinkeye"?** | | |
| The term "Pinkeye" refers to an infectious disease of cattle characterised by painful inflammation and ulceration of one or both eyes of the animal. Affected eyes typically show excessive tear secretion and a dull bluish-grey discoloration of the cornea (the transparent tissue in the centre of the eye) with or without a greyish-white ulcer. See pictures below: | | |
| 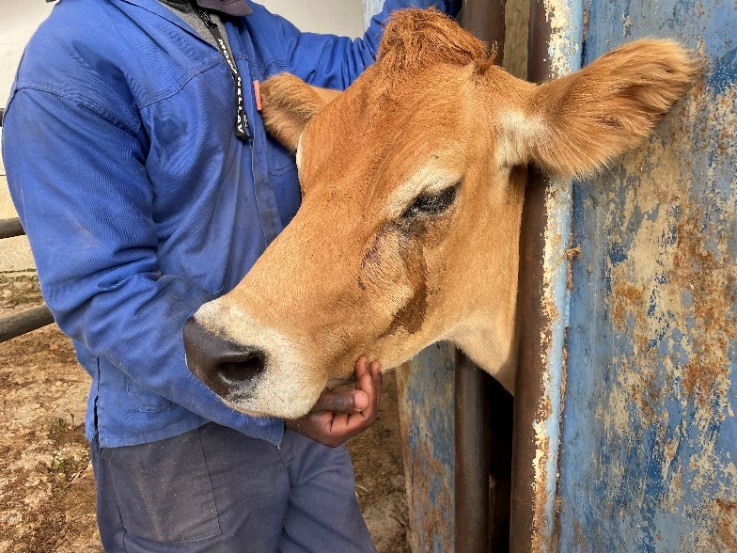 | 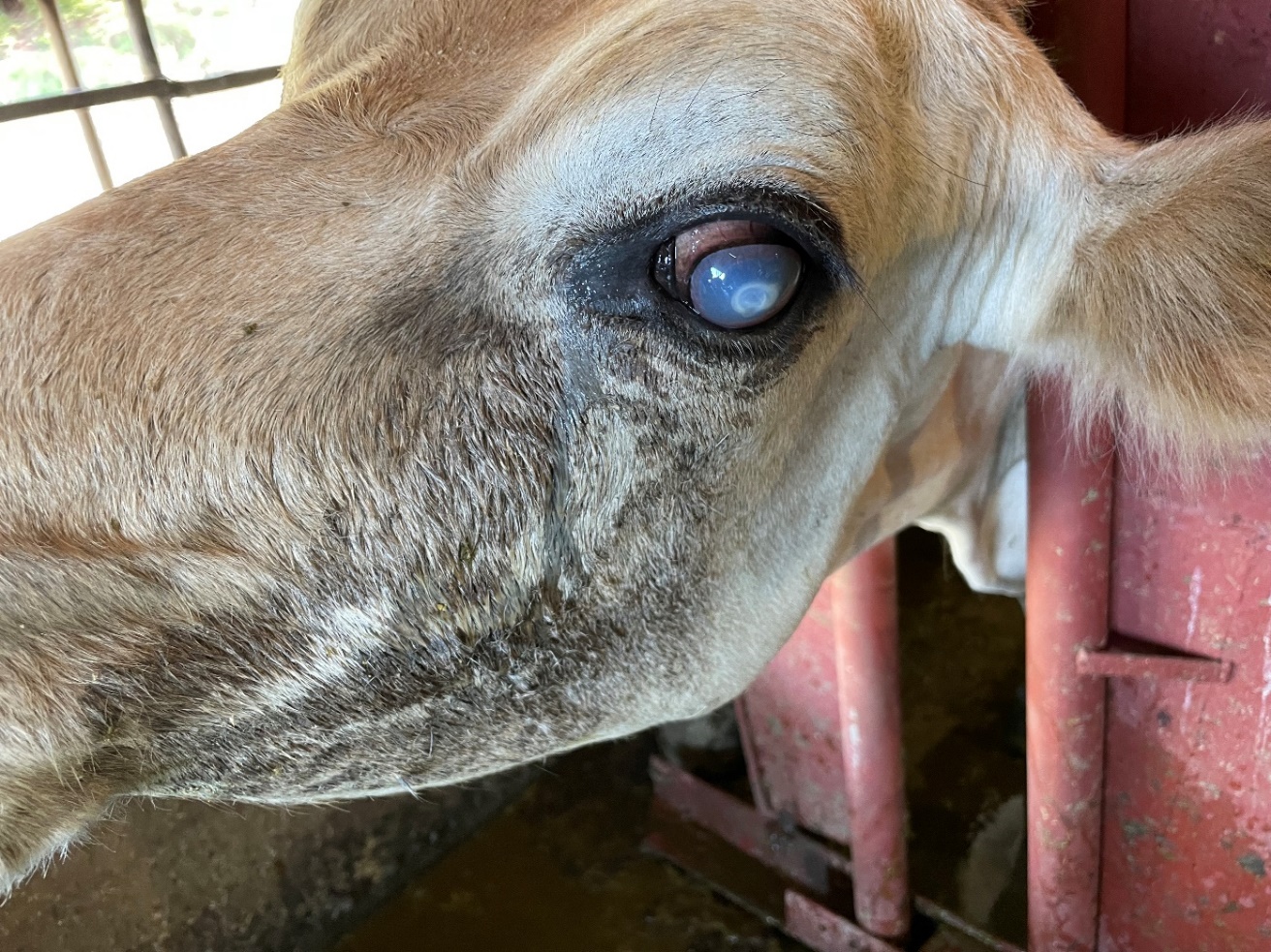 | 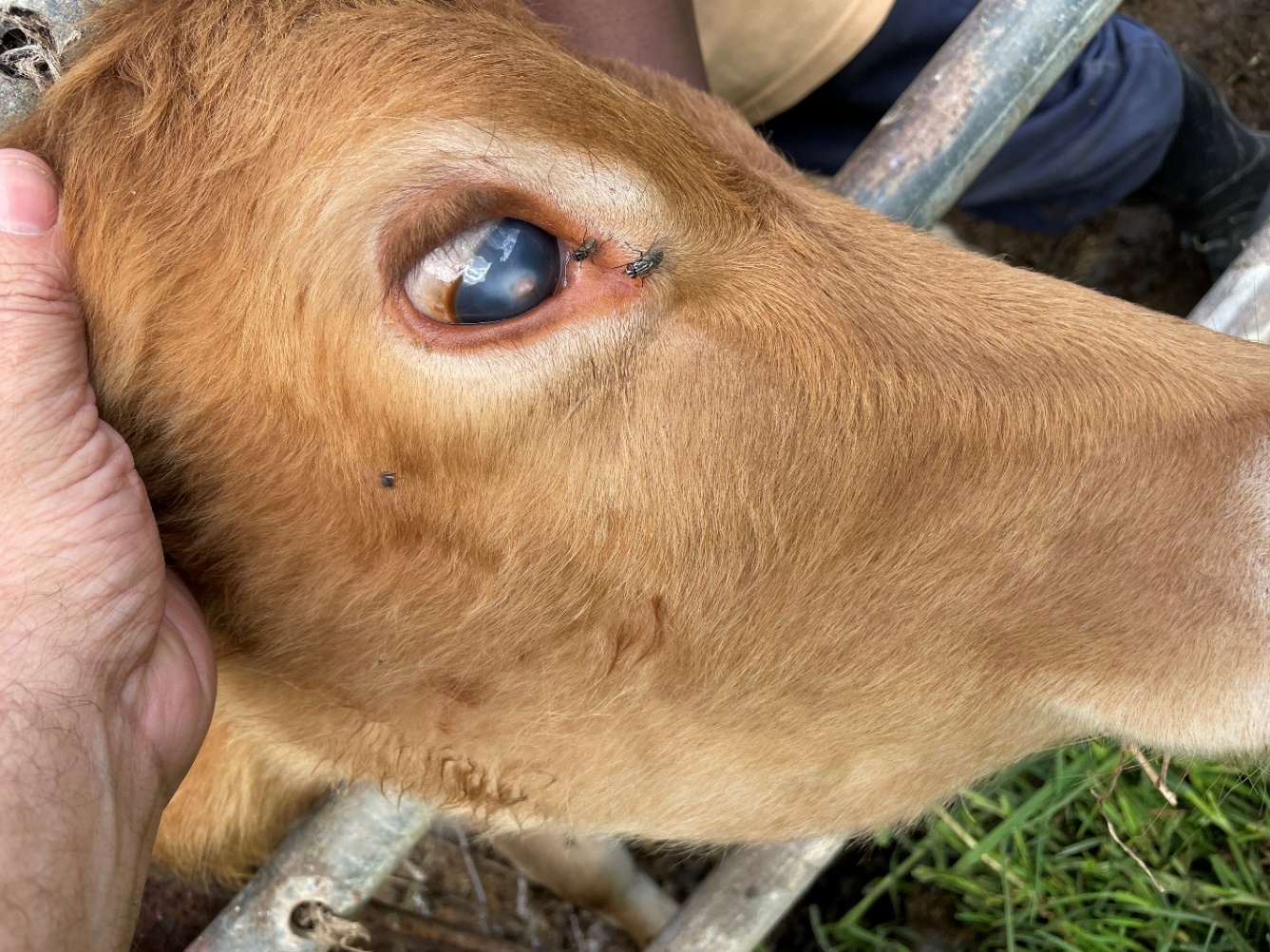 |

**PLEASE NOTE: All information will be treated as strictly confidential**

| **Farm ID:** |  | **Date of interview:** |  |
| --- | --- | --- | --- |
| **District:** |  | | |

### **GENERAL PERCEPTION OF THE DISEASE:**

| Have you seen pinkeye among your cattle during the last 5 years? | Yes | No |
| --- | --- | --- |

| Please categorise the intensity of pinkeye during each month of the year.(Very common = 3, Common = 2, Uncommon = 1, Generally absent = 0) | | | | | | | | | | | | | | | | |  |
| --- | --- | --- | --- | --- | --- | --- | --- | --- | --- | --- | --- | --- | --- | --- | --- | --- | --- |
| **Jan** | **Feb** | **Mar** | **Apr** | | **May** | **Jun** | | **Jul** | **Aug** | | **Sep** | **Oct** | | **Nov** | **Dec** | |  |
|  |  |  |  | |  |  | |  |  | |  |  | |  |  | |  |
| How common is pinkeye in the following age or production groups in your herd? | | | | | | | | | | | | | | | | | |
| **Age/Production group** | | | | **Not detected** | | | **Uncommon** | | | **Fairly common** | | | **Very common** | | | **I don’t know** | |
|  |  |  |  |  |  |  | (less than 1% affected) | | | (2% to 10% affected) | | | (more than 10% affected) | | |  |  |
| **Bulls** | | | |  | | |  | | |  | | |  | | |  | |
| **Lactating cows** | | | |  | | |  | | |  | | |  | | |  | |
| **Dry cows** | | | |  | | |  | | |  | | |  | | |  | |
| **First lactation heifers** | | | |  | | |  | | |  | | |  | | |  | |
| **Post-pubertal heifers** | | | |  | | |  | | |  | | |  | | |  | |
| **Pre-pubertal heifers** | | | |  | | |  | | |  | | |  | | |  | |
| **Pre-weaned calves** | | | |  | | |  | | |  | | |  | | |  | |

#### How much do you think the following factors contribute to pinkeye? Please rate them according to scale.

####

| **Factor** | **Do not contribute** | **Contribute, low importance** | **Contribute, moderate importance** | **Contribute, high importance** | **I don’t know** |
| --- | --- | --- | --- | --- | --- |
| **Build-up of manure** |  |  |  |  |  |
| **Dust from the environment** |  |  |  |  |  |
| **Dusty food (e.g. dry hay)** |  |  |  |  |  |
| **Eye-frequenting moths** |  |  |  |  |  |
| **Flies** |  |  |  |  |  |
| **Lack of shade** |  |  |  |  |  |
| **Long-stemmed grass** |  |  |  |  |  |
| **Newly introduced cattle** |  |  |  |  |  |
| **Rainfall, high (very wet conditions)** |  |  |  |  |  |
| **Rainfall, low (very dry conditions)** |  |  |  |  |  |
| **Season** |  |  |  |  |  |
| **Silage** |  |  |  |  |  |
| **Temperature, (average below 25°C)** |  |  |  |  |  |
| **Temperatures, (average above 25°C)** |  |  |  |  |  |
| **Ticks** |  |  |  |  |  |
| **UV irradiation** |  |  |  |  |  |
| **Wild antelope on the farm** |  |  |  |  |  |
| **Wind (high wind speed)** |  |  |  |  |  |
| **Wind (direction)** |  |  |  |  |  |

### **HERD COMPOSITION:**

| What breed of cattle do you currently have in your herd? (Please select from the list below and estimate the percentage of each breed in the herd). | | | | | | | | |
| --- | --- | --- | --- | --- | --- | --- | --- | --- |
|  | **Holstein** | **Jersey** | **Guernsey** | | **Ayrshire** | **Mixed breed** | **Other** | |
| **% of herd** |  |  |  | |  |  |  | |
| If “other” was selected, please name the breed(s)? | | | |  | | | |  |

| If you have more than one cattle breed(s) in your herd, in which breed do you see pinkeye most frequently? | | | | | | | |
| --- | --- | --- | --- | --- | --- | --- | --- |
| **Holstein** | **Jersey** | **Guernsey** | **Ayrshire** | | **Mixed breed** | **Other** | |
|  |  |  |  | |  |  | |
| If “other” was selected, please name the breed(s)? | | | |  | | |  |

| How many animals are in the herd and in each of the following age or production groups? | | | | | | |
| --- | --- | --- | --- | --- | --- | --- |
| **Lactating cows** | **Dry cows** | **First lactation heifers** | **Post-pubertal heifers** | **Pre-pubertal heifers** | **Pre-weaned calves** | **Total animals** |
|  |  |  |  |  |  |  |

| What is the average stocking density (animals/hectare) for the various categories of cattle in the camps or holding pens? | | | | |
| --- | --- | --- | --- | --- |
| **Lactating cows** | **Dry cows** | **First lactation heifers** | **Post-pubertal heifers** | **Pre-pubertal heifers** |
|  |  |  |  |  |

| Did you buy in any cattle during the past 5 years? | | | |  | Yes | | No |
| --- | --- | --- | --- | --- | --- | --- | --- |
| If “yes,” please provide the approximate numbers of cattle bought in during each year? | | | | | | | |
| **2022** | **2021** | **2020** | **2019** | | | **2018** | |
|  |  |  |  | | |  | |

### **NUTRITIONAL MANAGEMENT:**

| From the list below, select the type(s) of feed that are currently fed to heifers included in the study? (Please mark the correct option with a "✓") | | | | | | |
| --- | --- | --- | --- | --- | --- | --- |
| **Feeding strategy** | **Lactating cows** | **Dry cows** | | **First lactation heifers** | **Post pubertal heifers** | **Pre-pubertal heifers** |
| **Natural grazing** |  |  | |  |  |  |
| **Pastures (irrigated)** |  |  | |  |  |  |
| **Pastures (dry land)** |  |  | |  |  |  |
| **Silage** |  |  | |  |  |  |
| **Dry hay** |  |  | |  |  |  |
| **Crop residues** |  |  | |  |  |  |
| **Citrus by-products** |  |  | |  |  |  |
| **Apple pomace** |  |  | |  |  |  |
| **Brewer’s grain** |  |  | |  |  |  |
| **Grains (concentrates)** |  |  | |  |  |  |
| **Total mixed ration (TMR)** |  |  | |  |  |  |
| **Other** |  |  | |  |  |  |
| If “other” was selected, shortly describe this kind of feed: | | | | | | |
|  | | |  | | | |
|  | | |  | | | |

| Do you supply long stem roughage in hay racks to the cattle? | Yes | No |
| --- | --- | --- |
| Do you feed any concentrates in the parlour during milking? | Yes | No |
| Do you supplement vitamins (e.g. vitamin ADE) or minerals (e.g. | Yes | No |
| Multimin) by injection? |  |  |

|  | If you answered "yes", please list the injectable supplements currently used in the various age and production groups of the herd. | | | | | |  |
| --- | --- | --- | --- | --- | --- | --- | --- |
| **Injectable supplement** | | **Lactating cows** | **Dry cows** | **First lactation heifers** | **Post-pubertal heifers** | **Pre-pubertal heifers** | |
| a. | |  |  |  |  |  | |
| b. | |  |  |  |  |  | |
| c. | |  |  |  |  |  | |

### **GENERAL ANIMAL HUSBANDRY:**

| What type of flooring is available in the waiting enclosures around the milking parlour? | | | | |  |
| --- | --- | --- | --- | --- | --- |
| **Brick paving** | **Concrete** | **Gravel** | **Other** | | |
|  |  |  |  | | |
| If “other” was selected, shortly describe this type of flooring: | | | |  | |
|  | | | | | |
|  | | | | | |

| How often do you remove manure from the waiting enclosures around the milking parlour? | | | | | |
| --- | --- | --- | --- | --- | --- |
| **After every milking** | **Daily** | **Weekly** | **Monthly** | **Other** | **Never** |
|  |  |  |  |  |  |
| If “other” was selected, describe the cleaning frequency of the enclosures around the parlour. | | | | | |
|  | | | | | |
|  | | | | | |

| How often do you remove manure from the kraals and holding pens? | | | | | |
| --- | --- | --- | --- | --- | --- |
| **Weekly** | **Monthly** | **Every 2 - 6 months** | **Every 6 - 12 months** | **Other** | |
|  |  |  |  |  | |
| If “other” was selected, shortly describe how often do you clean the kraals and holding pens. | | | | | |
|  | | | | |  |
|  | | | | |  |

#### Please estimate the percentage surface area under shade in the holding pens for each category of animal?

If you answered “yes”, please tick the applicable type of shade for each age or production group.

| **Age/Production group** | **% surface area under shade** |  | **Type of shade** | | | |
| --- | --- | --- | --- | --- | --- | --- |
|  |  |  | **Trees** | **Shade cloth** | **Corrugated iron roof** | **Other** |
| **Lactating cows** |  |  |  |  |  |  |
| **Dry cows** |  |  |  |  |  |  |
| **First lactation heifers** |  |  |  |  |  |  |
| **Post-pubertal heifers** |  |  |  |  |  |  |
| **Pre-pubertal heifers** |  |  |  |  |  |  |
| **Pre-weaned calves** |  |  |  |  |  |  |
| If “other” was selected, shortly describe how the shade is created (*e.g.* what material is used to create shade, etc.). | | | | | | |
|  | | | | | | |
|  | | | | | | |

| \| - - 1. Please categorise the fly burden on your farm for each month of the year.  *(Very high = 3, High = 2, Low = 1, Extremely low or absent = 0)* \| \| \| \| \| \| \| \| \| \| \| \| \| --- \| --- \| --- \| --- \| --- \| --- \| --- \| --- \| --- \| --- \| --- \| --- \| \| **Jan** \| **Feb** \| **Mar** \| **Apr** \| **May** \| **Jun** \| **Jul** \| **Aug** \| **Sep** \| **Oct** \| **Nov** \| **Dec** \| \|  \|  \|  \|  \|  \|  \|  \|  \|  \|  \|  \|  \| \|  \|  \|  \|  \|  \|  \|  \|  \|  \|  \|  \|  \|  \| Do you have a fly control programme? \| Yes \| No \| \| --- \| --- \| --- \| \|  \|  \|  \|  \| If you answered "yes", please tick the applicable control measures. \| \| \| \| \| \| --- \| --- \| --- \| --- \| --- \| \| **Insect growth regulators (e.g. Larvadex)** \| **Parasitic wasps** \| **Insecticidal sprays (environment)** \| **Insecticidal products (on animal)** \| **Other** \| \| \|  \|  \|  \|  \|  \| \| \| If “other” was selected, shortly describe the control measure. \| \| \| \| \| \| \|  \| \| \| \| \| \|  \| Please categorise the dust levels on your farm for each month of the year. *(Very high = 3, High = 2, Low = 1, Extremely low or absent = 0)* \| \| --- \|  \| **Jan** \| **Feb** \| **Mar** \| **Apr** \| **May** \| **Jun** \| **Jul** \| **Aug** \| **Sep** \| **Oct** \| **Nov** \| **Dec** \| \| --- \| --- \| --- \| --- \| --- \| --- \| --- \| --- \| --- \| --- \| --- \| --- \| \|  \|  \|  \|  \|  \|  \|  \|  \|  \|  \|  \|  \|  Do you associate any camp or holding area on your farm with a higher frequency of  \|  \| pinkeye among the cattle? \| \| \| Yes \| \| No \| \| \| --- \| --- \| --- \| --- \| --- \| --- \| --- \| --- \| \| If you answered “yes,” please answer the following questions about the conditions in the camp showing a higher pinkeye frequency. Also indicate if these conditions are similar, or different, compared to the other camps. \| \| \| \| \| \| \| \| \| **Factors in the camp or holding pen associated with a higher frequency of pinkeye** \| \| **More** \| **Similar** \| \| **Less** \| \| **Don’t know** \| \| Is the stocking density in this camp or holding pen higher, lower, or like the other areas on the farm? \| \|  \|  \| \|  \| \|  \| \| Do you feed dusty food (e.g. dry hay) in this camp or holding area more frequently, or less frequently, compared to other areas on the farm? Or are the feeding practices in this camp like other areas on the farm? \| \|  \|  \| \|  \| \|  \| \| Is there more, or less, long-stemmed grass in this camp or holding pen compared to other areas on the farm? Or is the amount of long-stemmed grass in this camp like other areas of the farm? \| \|  \|  \| \|  \| \|  \| \| Do you find eye-frequenting moths more often, or less often, in this camp or holding pen compared to other areas on the farm? Or do you find these moths with a similar frequency in all areas of the farm? \| \|  \|  \| \|  \| \|  \| \| Are there more flies, less flies, or similar amounts of flies in this camp or holding area compared to other areas on the farm? \| \|  \|  \| \|  \| \|  \| \| Is the amount of manure that accumulated in this camp or holding pen more, or less, compared to other areas on the farm? Or is the amount of accumulated manure like the other areas on the farm? \| \|  \|  \| \|  \| \|  \| \| Is the area covered by shade in this camp or holding pen larger, smaller, or similar compared to other areas on the farm? \| \|  \|  \| \|  \| \|  \| \| Does this camp or holding area have better drainage, poorer drainage or similar drainage compared to the other areas on the farm? \| \|  \|  \| \|  \| \|  \| \| Is this camp or holding pen situated at a higher, lower, or similar altitude compared to the other areas on the farm? \| \|  \|  \| \|  \| \|  \| \| Are the animals in this camp or holding pen more exposed, or less exposed, to windy conditions? Or is the wind exposure in this camp or holding pen like other areas on the farm? \| \|  \|  \| \|  \| \|  \| \| Is there more, or less, wild antelope in this camp or holding pen compared to other areas on the farm? Or is the number of wild antelope in this camp like other areas on the farm? \| \|  \|  \| \|  \| \|  \|  Can you think of any factor(s), other than the factors already mentioned in the table of Question 1.4.8, that is different between the camp or holding pen with a greater frequency of disease and the other areas on the farm?  \|  \| Yes \| No \| \| --- \| --- \| --- \|  \| If you answered "yes", please name this factor(s). \| \| --- \| \|  \| \| \|  \| \| |
| --- | --- | --- | --- | --- | --- | --- | --- | --- | --- | --- | --- | --- | --- | --- | --- | --- | --- | --- | --- | --- | --- | --- | --- | --- | --- | --- | --- | --- | --- | --- | --- | --- | --- | --- | --- | --- | --- | --- | --- | --- | --- | --- | --- | --- | --- | --- | --- | --- | --- | --- | --- | --- | --- | --- | --- | --- | --- | --- | --- | --- | --- | --- | --- | --- | --- | --- | --- | --- | --- | --- | --- | --- | --- | --- | --- | --- | --- | --- | --- | --- | --- | --- | --- | --- | --- | --- | --- | --- | --- | --- | --- | --- | --- | --- | --- | --- | --- | --- | --- | --- | --- | --- | --- | --- | --- | --- | --- | --- | --- | --- | --- | --- | --- | --- | --- | --- | --- | --- | --- | --- | --- | --- | --- | --- | --- | --- | --- | --- | --- | --- | --- | --- | --- | --- | --- | --- | --- | --- | --- | --- | --- | --- | --- | --- | --- | --- | --- | --- | --- | --- | --- | --- | --- | --- | --- | --- | --- | --- | --- | --- | --- | --- | --- | --- | --- | --- | --- | --- | --- | --- | --- | --- | --- | --- | --- | --- | --- | --- | --- | --- | --- | --- | --- | --- | --- | --- | --- | --- | --- | --- | --- | --- | --- | --- | --- | --- | --- | --- | --- | --- | --- | --- | --- | --- | --- | --- | --- | --- | --- | --- | --- | --- | --- | --- | --- | --- | --- | --- | --- | --- | --- | --- | --- | --- | --- | --- | --- | --- |

### **VACCINATION PROGRAMME**

| Do you vaccinate the herd against pinkeye? | | | | Yes | No |
| --- | --- | --- | --- | --- | --- |
| If you answered "yes", name the vaccine(s) you are using for each age or production group in the herd. | | | | | |
| **Age/Production group** | **Name of vaccine** |  | **How often do you vaccinate** | | |
| **Lactating cows** |  |  |  | | |
| **Dry cows** |  |  |  | | |
| **First lactation heifers** |  |  |  | | |
| **Post-pubertal heifers** |  |  |  | | |
| **Pre-pubertal heifers** |  |  |  | | |
| **Pre-weaned calves** |  |  |  | | |

| Do you vaccinate the herd against pneumonia or “pasteurellosis”? | | | | Yes | No | |  |
| --- | --- | --- | --- | --- | --- | --- | --- |
| If you answered "yes", name the vaccine(s) you are using for each age or production group. | | | | | | | |
| **Age/Production group** | **Name of vaccine** |  | **How often do you vaccinate** | | |  |  |
| **Lactating cows** |  |  |  | | |  |  |
| **Dry cows** |  |  |  | | |  |  |
| **First lactation heifers** |  |  |  | | |  |  |
| **Post-pubertal heifers** |  |  |  | | |  |  |
| **Pre-pubertal heifers** |  |  |  | | |  |  |
| **Pre-weaned calves** |  |  |  | | |  |  |

| Do you vaccinate the herd against bovine viral diarrhea (BVD)? | | | | Yes | No | |
| --- | --- | --- | --- | --- | --- | --- |
| If you answered "yes", name the vaccine(s) you are using for each age or production group. | | | | | |  |
| **Age/Production group** | **Name of vaccine** |  | **How often do you vaccinate** | | | |
| **Lactating cows** |  |  |  | | | |
| **Dry cows** |  |  |  | | | |
| **First lactation heifers** |  |  |  | | | |
| **Post-pubertal heifers** |  |  |  | | | |
| **Pre-pubertal heifers** |  |  |  | | | |
| **Pre-weaned calves** |  |  |  | | | |

**Acknowledgements:**

We gratefully acknowledge M. Kneipp and the University of Sydney, NSW, Australia for questions derived from the *SurveyMonkey* questionnaire "Beef Producer Pinkeye Survey".

**ONLINE RESOURCE:**

**Supplementary figures**

| 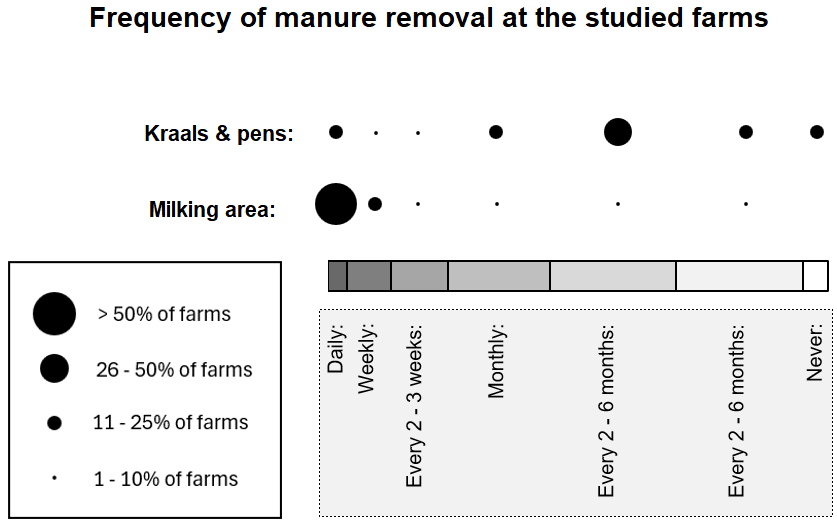 |
| --- |
| Figure S1: Manure removal routines encountered on the studied farms. Most farmers hosed the waiting areas around the milking parlour after every milking or daily, while manure was removed holding pens and kraals at intervals of 2 to 6 months. |

| **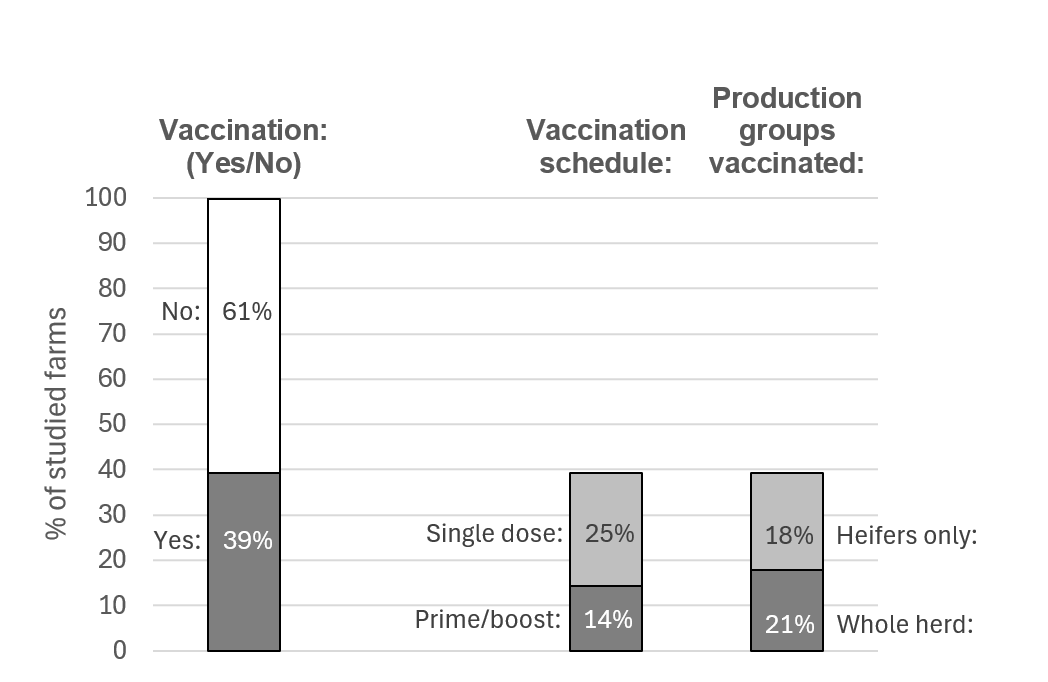** |
| --- |
| **Figure S2:** The proportions of the participating farms that routinely vaccinated against infectious bovine keratoconjunctivitis. From the ~39% of farms that vaccinated, 25% administered a single dose of the vaccine during spring, while 14% of the farms followed a prime/boost approach. Eighteen percent of the vaccinating farms limited vaccine administration to heifers, while 21% vaccinated all age groups (which implies yearly re-vaccination of the older cattle). |

**ONLINE RESOURCE**

**Supplementary Tables**

| **Table S1: Breed composition (%) in the Western Cape as estimated by the MPO, in comparison to the breed composition on the sampled farms and the sampled cattle.** |
| --- |

|  | MPO estimate for the Western Cape Province (%) | Sampled farms (%)  (n = 28) | Sampled cattle (%)  (n = 1675) |
| --- | --- | --- | --- |
| Holstein | 58 | 52 | 24 |
| Jersey | 36 | 39 | 56 |
| Ayrshire | 5 | 7 | 15 |
| Guernsey | 1 | 1 | 2 |
| Mixed breed |  | 1 | 2 |

| **Table S2: Primer and probe sequences utilised for the detection of *Moraxella bovis*, *Moraxella bovoculi*, *Mesomycoplasma bovoculi* and *Mycoplasmopsis bovis* in the multiplex real-time PCR on the Roche LightCycler® Nano.** | | | | |
| --- | --- | --- | --- | --- |
| **Reagent** | **Sequence** | | **Amplicon size (bp)** | |
| **Moraxella bovis** |  | | | 63 |
| Forward primer | **GGTGACGACCGCTTGTTT** | | |  |
| Reverse primer | **ATCATCGCCTTCATCTCCAG** | | |  |
| Hydrolysis probe | **6FAM-CGATCGTTGCCTTTACCACC--BHQ1** | | |  |
| **Moraxella bovoculi** |  | | | 90 |
| Forward primer | **GGTGATATTTATCATGAAGTTGTGAAA** | | |  |
| Reverse primer | **TYTCAATTCATAATCACGATACTCAAG** | | |  |
| Hydrolysis probe | **LC610-CCAAGATACTGCGGTAGGTAAACG--BBQ’** | | |  |
| **Mycoplasmopsis bovis** |  | | | 110 |
| Forward primer | **GCTGATGGCGGTATACAACA** | | |  |
| Reverse primer | **GCTTTGGTTTTGTGAAACTC** | | |  |
| Hydrolysis probe | **6FAM-CGCTTAAAACGCTTAATATAAACATCC--BHQ1** | | |  |
| **Mesomycoplasma bovoculi** | |  | | 66 |
| Forward primer | **AGCTATGGCAGGGGACAAC** | | |  |
| Reverse primer | **CCACGTTCAATGTCTTTACGG** | | |  |
| Hydrolysis probe | **LC610-CTGGTGTTCTACTCCGTGGTATTG--BBQ** | | |  |

**Table S3: Prevalence of bovine keratoconjunctivitis (BK) and the percentage of eyes testing (+) for the various microbial factors, grouped by cluster.**

| **Farm** | **BK prevalence**  **at farm level** |  | **Proportion of cattle eyes testing (+) for:** | | | |
| --- | --- | --- | --- | --- | --- | --- |
|  |  |  | ***Mor.***  ***bovis*** | ***Mor. bovoculi*** | ***Mes. bovoculi*** | ***Myc.***  ***bovis*** |
| Farm 01 | 8.5 |  | - | 35.6 | 76.3 | - |
| Farm 02 | 1.7 |  | - | - | 45.8 | - |
| Farm 03 | 26.7 |  | 30.0 | 10.0 | 81.7 | - |
| Farm 04 | 23.3 |  | 8.3 | 3.3 | 93.3 | - |
| Farm 05 | 13.6 |  | 23.7 | 3.4 | 94.9 | - |
| Farm 06 | 16.7 |  | 8.3 | - | 76.7 | - |
| Farm 07 | 11.7 |  | 43.3 | - | 81.7 | 1.7 |
| Farm 08 | 18.3 |  | 8.3 | - | 81.7 | - |
| Farm 09 | 23.3 |  | 16.7 | - | 90.0 | - |
| Farm 10 | 13.3 |  | 8.3 | 6.7 | 76.7 | - |
| Farm 11 | 35.0 |  | 20.0 | 11.7 | 85.0 | - |
| Farm 12 | 15.0 |  | 6.7 | - | 75.0 | 1.7 |
| Farm 13 | 10.0 |  | 5.0 | 5.0 | 56.7 | - |
| Farm 14 | 25.0 |  | 25.0 | 5.0 | 58.3 | - |
| Farm 15 | 8.3 |  | 5.0 | 20.0 | 46.7 | - |
| Farm 16 | 13.3 |  | 11.7 | 6.7 | 65.0 | 3.3 |
| Farm 17 | 18.6 |  | 10.2 | - | 91.5 | - |
| Farm 18 | 20.0 |  | 11.7 | 5.0 | 86.7 | - |
| Farm 19 | 20.3 |  | 5.1 | 45.8 | 88.1 | - |
| Farm 20 | 23.3 |  | 16.7 | 21.7 | 86.7 | - |
| Farm 21 | 8.3 |  | 26.7 | 8.3 | 76.7 | - |
| Farm 22 | 33.3 |  | 46.7 | 28.3 | 88.3 | - |
| Farm 23 | 5.0 |  | - | - | 85.0 | - |
| Farm 24 | 23.3 |  | 6.7 | 6.7 | 93.3 | - |
| Farm 25 | 10.0 |  | 25.0 | 6.7 | 95.0 | 1.7 |
| Farm 26 | 10.0 |  | 8.3 | - | 96.7 | 1.7 |
| Farm 27 | 0.0 |  | 8.3 | - | 95.0 | - |
| Farm 28 | 15.0 |  | 46.7 | 1.7 | 93.3 | - |
| Abbreviations: Mor. = *Moraxella*, Mes. = *Mesomycoplasma*, Myc. = *Mycoplasmopsis* | | | | | | |

| **Table S4: Univariate analysis of the associations between bovine keratoconjunctivitis (BK) and potential host, environmental and agent risk factors.** | | | | | | | | |
| --- | --- | --- | --- | --- | --- | --- | --- | --- |
| **Variables and category** | **Cattle exposed** | | | | **Cattle with BK** | **Odds ratio** | **95% CI** | ***P*-value** |
| Breed: |  | | | |  |  |  | < 0.01^a^ |
| Holstein | 405 | | | | 88 | 1.9 | 1.4 – 2.5 |  |
| Jersey | 944 | | | | 122 | (base) |  |  |
| Guernsey | 39 | | | | 5 | 1.0 | 0.4 – 2.6 |  |
| Ayrshire | 256 | | | | 47 | 1.5 | 1.0 – 2.2 |  |
| Mixed breed | 31 | | | | 8 | 1.8 | 1.0 – 5.4 |  |
| Production group: |  | | | |  |  |  | < 0.01^a^ |
| Lactating cows | 727 | | | | 92 | 1.2 | 0.6 – 2.3 |  |
| Dry cows | 112 | | | | 12 | (base) |  |  |
| First lactation heifers | 223 | | | | 39 | 1.8 | 0.9 – 3.5 |  |
| Post-pubertal heifers | 148 | | | | 29 | 2.0 | 0.9 – 4.2 |  |
| Pre-pubertal heifers | 356 | | | | 68 | 2.0 | 1.0 – 3.8 |  |
| Pre-weaned calves | 109 | | | | 30 | 3.2 | 1.5 – 6.6 |  |
| Sampling month: |  | | | |  |  |  | 0.08^a^ |
| January | 238 | | | | 36 | 0.8 | 0.5 – 1.2 |  |
| February | 359 | | | | 58 | 0.9 | 0.6 – 1.2 |  |
| March | 778 | | | | 141 | (base) |  |  |
| April | 300 | | | | 35 | 0.6 | 0.4 – 0.9 |  |
| State vet. District: |  | | | |  |  |  | 0.13^a^ |
| Boland | 179 | | | | 21 | 0.7 | 0.4 – 1.1 |  |
| Malmesbury | 299 | | | | 59 | 1.3 | 0.9 – 1.8 |  |
| Swellendam | 958 | | | | 154 | (base) |  |  |
| Worcester | 239 | | | | 36 | 0.9 | 0.6 – 1.4 |  |
| Lower body condition score (≤ 2) | 364 | | | | 60 | 1.0 | 0.8 – 1.4 | 0.83 ^a^ |
| Animals in lactation | | 950 | | | 131 | 0.7 | 0.5 – 0.9 | < 0.01^a^ |
| High dust levels 1 month before sampling | | 898 | | | 164 | 1.4 | 1.1 – 1.8 | 0.01^a^ |
| High dust levels during month of sampling | | | 598 | | 118 | 1.5 | 1.1 – 1.9 | < 0.01^a^ |
| High fly burden 1 month before sampling | | 959 | | | 169 | 1.3 | 1.0 – 1.7 | 0.05^a^ |
| High fly burden during month of sampling | | | 599 | | 128 | 1.8 | 1.4 – 2.3 | < 0.01^a^ |
| Fly control by backyard chickens | 120 | | | | 6 | 0.3 | 0.1 – 0.6 | < 0.01^a^ |
| Fly control by insect growth regulators | 360 | | | | 75 | 1.5 | 1.1 – 2.0 | 0.01^a^ |
| Fly control by pesticides | 1377 | | | | 237 | 1.7 | 1.1 – 2.5 | 0.01^a^ |
| Fly control by parasitic wasps | 119 | | | | 9 | 0.4 | 0.2 – 0.8 | 0.01^a^ |
| Effluent processing & composting plant | | 120 | | | 18 | 0.9 | 0.5 – 1.5 | 0.73^a^ |
| Multi-component fly control (≥ 3 elements) | | | 599 | | 96 | 1.0 | 0.8 – 1.3 | 0.94^a^ |
| No fly control programme | 59 | | | | 5 | 0.5 | 0.2 – 1.2 | 0.15^b^ |
| Ration containing apple pomace | 231 | | | | 29 | 0.7 | 0.5 – 1.1 | 0.11^a^ |
| Ration containing brewer’s grain | 70 | | | | 14 | 1.3 | 0.7 – 2.4 | 0.37^a^ |
| Ration containing citrus residue | 461 | | | | 60 | 0.7 | 0.5 – 1.0 | 0.03^a^ |
| Ration containing silage | 733 | | | | 98 | 0.7 | 0.5 – 0.9 | 0.01^a^ |
| Ration containing dry hay | 1087 | | | | 178 | 0.6 | 0.5 – 0.8 | < 0.01^a^ |
| Ration containing grains (meal or pellets) | | | 1034 | | 145 | 0.7 | 0.5 – 0.9 | < 0.01^a^ |
| Total mixed ration | 706 | | | | 113 | 1.0 | 0.8 – 1.3 | 0.91^a^ |
| Grazing on irrigated pastures | 832 | | | | 123 | 0.8 | 0.6 – 1.1 | 0.14^a^ |
| Grazing on dryland pastures | 574 | | | | 90 | 1.0 | 0.7 – 1.3 | 0.72^a^ |
| Utilising natural grazing | 245 | | | | 39 | 1.0 | 0.7 – 1.4 | 0.93^a^ |
| Utilising crop residues | 249 | | | | 31 | 0.7 | 0.5 – 1.1 | 0.09^a^ |
| Larger herds (≥ 874) | 838 | | | | 151 | 1.3 | 1.0 – 1.7 | 0.03^a^ |
| Higher stocking density (≥ 40 animals/ha) | | | 359 | | 71 | 1.4 | 1.0 – 1.9 | 0.03^a^ |
| Stable herd size during last 5 years | 1017 | | | | 145 | 0.7 | 0.5 – 0.9 | 0.01^a^ |
| Cattle introductions during the last 5 years | | | 419 | | 89 | 1.6 | 1.2 – 2.1 | < 0.01^a^ |
| Cleaning frequency, milking area: ≤ 7 days | | | 1436 | | 251 | 2.5 | 1.5 – 4.0 | < 0.01^a^ |
| Cleaning frequency, holding pens: ≤ 7 days | | | | 239 | 20 | 0.4 | 0.3 – 0.7 | < 0.01^a^ |
| Gravel flooring around milking parlour | 418 | | | | 56 | 0.8 | 0.5 – 1.0 | 0.08^a^ |
| Insufficient shade in camps | 756 | | | | 147 | 1.6 | 1.2 – 2.0 | < 0.01^a^ |
| Access to dappled shade under trees | 533 | | | | 72 | 0.7 | 0.6 – 1.0 | 0.05^a^ |
| Access to semi-shade under shade cloth | | 153 | | | 29 | 1.2 | 0.8 – 1.9 | 0.32^a^ |
| Access to solid shade under roofs | | 600 | | | 97 | 1.0 | 0.8 – 1.3 | 0.97^a^ |
| Vaccinating against pinkeye (heifers only) | | | 299 | | 61 | 1.4 | 1.0 – 2.0 | 0.03^a^ |
| Vaccinating against pinkeye (whole herd) | | 658 | | | 108 | 1.0 | 0.8 – 1.4 | 0.79^a^ |
| Vaccination against BRD (whole herd) | | 956 | | | 161 | 1.1 | 0.9 – 1.5 | 0.35^a^ |
| Using inactivated BHV1 & BVD vaccines | | 538 | | | 100 | 1.3 | 1.0 – 1.7 | 0.06^a^ |
| Using live BVD vaccines | 538 | | | | 69 | 0.7 | 0.5 – 0.9 | 0.01^a^ |
| Using live BHV1 vaccines | 837 | | | | 135 | 1.0 | 0.8 – 1.3 | 0.99^a^ |
| *Mor. bovis* present | 259 | | | | 56 | 1.5 | 1.1 – 2.2 | 0.01^a^ |
| *Mor. bovoculi* present | 138 | | | | 40 | 2.3 | 1.6 – 3.4 | < 0.01^a^ |
| *Mes. bovoculi* present | 1353 | | | | 239 | 2.0 | 1.4 – 3.0 | < 0.01^a^ |
| *Myc. bovis present* | 6 | | | | 1 | 1.0 | 0.1 – 8.9 | 1.00^b^ |
| Abbreviations: BK: Bovine keratoconjunctivitis; BRD: Bovine respiratory disease; BHV1: Bovine herpesvirus type 1; BVD: Bovine viral diarrhoea.  ^a^ *P*-value derived from Chi-squared analysis.  ^b^ *P*-value derived from Fisher’s exact test. | | | | | | | | |
